# Clinical Outcomes of Primary Palatal Surgery in Children with Nonsyndromic Cleft Palate with and without Lip

**DOI:** 10.1155/2015/185459

**Published:** 2015-07-27

**Authors:** Seunghee Ha, Kyung S. Koh, Heewon Moon, Seungeun Jung, Tae Suk Oh

**Affiliations:** ^1^Division of Speech Pathology and Audiology, Audiology and Speech Pathology Research Institute, Hallym University, Chuncheon 200-702, Republic of Korea; ^2^Department of Plastic Surgery, Seoul Asan Medical Center, University of Ulsan College of Medicine, Seoul 138-736, Republic of Korea

## Abstract

This study presents clinical outcomes of primary cleft palate surgery, including rate of oronasal fistula development, rate of velopharyngeal insufficiency (VPI) requiring secondary surgery, and speech outcomes. We examined the effect of cleft type on the clinical outcomes. Retrospective analysis was performed using clinical records of all patients who received a primary palatoplasty at the Cleft Palate Clinic at Seoul Asan Medical Center, South Korea, between 2007 and 2012. The study included 292 patients with nonsyndromic overt cleft palate (±cleft lip). The results revealed that the rate of oronasal fistula was 7.9% and the incidence of VPI based on the rate of secondary palatal surgery was 19.2%. The results showed that 50.3% of all the patients had received speech therapy and 28.8% and 51.4% demonstrated significant hypernasality and articulatory deficits, respectively. The results of the rate of VPI and speech outcomes were significantly different in terms of cleft type. Except for the rate of oronasal fistula, patients with cleft palate generally exhibited better clinical outcomes compared to those with bilateral or unilateral cleft lip and palate. This study suggests that several factors, including cleft type, should be identified and comprehensively considered to establish an optimal treatment regimen for patients with cleft palate.

## 1. Introduction

Cleft palate is the most common type of innate craniofacial anomaly, which requires multidisciplinary treatment approach, including physical palatal correction, feeding management, orthodontic management, and speech-language services. Primary surgical correction of the cleft palate is typically performed by 12 months of age, and it ultimately aims to restore a mechanism for normal speech production. The criteria for successful primary palatal surgery include rates of occurrence of oronasal fistula, rates of persistent velopharyngeal insufficiency (VPI), and the achievement of normal speech. Over the past several years, advances have been made in surgical management of cleft palate in terms of surgical techniques and timing of palatal surgery [1–5], which has decreased the postoperative rate of oronasal fistula, decreased the rate of persistent VPI, and improved speech outcomes. Surgical palatal techniques have focused on a proper muscle repair (e.g., Furlow double-opposing Z-plasty), and the timing of palatal surgery has dramatically decreased from 18–24 months before the 1980s to 9–12 months in the present [6]. These changes in surgical management of cleft palate have led to improved clinical outcomes.

A number of studies reviewed cleft palate management employed by various centers worldwide. Clinical outcomes following primary palatal surgery tend to vary significantly, although the results have generally improved compared to those in the past. The incidence of oronasal fistula has been documented to range from 0% to 12.8% in the recent studies [5, 7–12]. A meta-analysis using 11 studies published between 2000 and 2012 comprising 2505 children found that the rate of fistula formation following primary palatal surgery was 4.9% [11]. The study also reported that patients with complete bilateral clefts (Veau IV) would be more likely to develop a fistula. The reports of incidence of persistent VPI following primary surgery vary across the literature, with some studies reporting incidence as high as 30% [5–7, 13–15]. In general, the rate of VPI reportedly decreased in the condition using surgical palatal techniques with emphasis on a proper muscle, although the rate was higher in patients with more severe and wider clefts [7]. The relationship between the rate of VPI and cleft type was not statistically significant in some studies [5, 7, 16]. However, some other studies reported that VPI is more frequent in cleft lip and palate than in isolated cleft palate [16].

Furthermore, the existing studies reported a wide range of speech outcomes following primary surgery in terms of different speech aspects and assessment methods. Most studies reported speech outcomes based on the presence or severity of hypernasal speech. Some studies also reviewed, in detail, articulatory issues that negatively affected many children with cleft palate. It is reported that up to approximately 25% to 30% of the children with cleft palate still tend to exhibit speech problems throughout most of their important formative preschool age and school-age years [17]. Hardin-Jones and Jones (2005) found that 37% of 212 preschoolers with repaired cleft palate demonstrated significant hypernasality or received secondary surgical management for velopharyngeal insufficiency. The study also reported that approximately two-thirds of the children demonstrated significant speech production problems and therefore were enrolled in direct speech therapy. In addition, the study indicated that a smaller percentage of children with clefts of the soft palate required speech therapy for articulatory problems and showed significant hypernasality compared to children with bilateral or unilateral cleft lip and palate and clefts of the hard and soft palate [6].

It is important to evaluate clinical outcomes of primary palatal surgery and to identify factors related to clinical outcomes in order to improve cleft care and achieve the ultimate goal of individuals with cleft palate, that is, to restore a mechanism for normal speech production. The purpose of the study was to investigate the clinical outcomes of primary palatal surgery in patients with nonsyndromic overt cleft palate (±cleft lip) treated in the cleft palate-craniofacial clinic of the Seoul Asan Medical Center, South Korea. The study presents an analysis of the results of cleft palate surgery, including postoperative rate of oronasal fistula development, rate of VPI necessitating secondary surgery, and speech outcomes. Moreover, we evaluate the effect of the cleft type on the clinical outcomes.

## 2. Materials and Methods

### 2.1. Participants

The Seoul Asan Medical Center Institutional Review Board approved this study. The study involved a retrospective analysis of 459 patients who received primary palatal surgery in the Cleft Palate-Craniofacial Clinic of the Seoul Asan Medical Center, South Korea, between 2007 and 2012. All patients had received primary palatal surgery performed by one plastic surgeon (the second author, K. S. Koh) in the clinic. Primary palatal surgery in the clinic is implemented at around 12 months of age using a Furlow double opposing Z-plasty, two-flap palatoplasty, intravelar veloplasty, or von Langenbeck flaps. A two-stage approach (soft palate closure at 9–12 months of age and hard palate closure around 18 months of age) has also been performed in some cases involving wide clefting of the palate. Choice of primary palatal surgical techniques was based on preoperative cleft anatomy. In addition, an orthodontist, a member of the cleft palate team at the medical center who works at a local dental clinic outside of the medical center, treated most babies born with unilateral or bilateral cleft lip and palate with preoperative nasoalveolar molding (NAM).

For inclusion in the current study, patients should be born with overt cleft palate, and they must have been seen for routine follow-up speech examinations at least until 36 months of age or for two years from the time of primary palatoplasty. Patients with submucous cleft palate were excluded from this study. None of the patients had demonstrated syndrome, other congenital anomalies, sensorineural hearing impairment, cognitive deficits, or neurological involvement. Two hundreds ninety-two patients with nonsyndromic overt cleft palate finally met the inclusion criteria. Of 292 patients in total, 41 patients had unilateral cleft lip and palate (UCLP), 94 had bilateral cleft lip and palate (BCLP), and 157 had clefts of palate only (CPO).

### 2.2. Procedures

The data on cleft type, sex, age at palatal surgery, and postoperative complications (e.g., oronasal fistula, VPI), along with secondary palatal surgery rates and the results of follow-up speech examinations, were obtained from patients' electronic medical records. This study included only clinically significant oronasal fistula developed at the anterior part of the hard palate (i.e., the hard palate anterior or posterior to incisive foramen) and in the region at the junction of the soft and hard palate according to the medical records. The follow-up speech examinations were administered to each participant at 12 (one month after primary palatoplasty), 15, 24, and 36 months of age. For the purpose of this study, the data from the 36-month follow-up examination were considered as clinical decisions (i.e., speech therapy and/or secondary palatal surgery, or termination of routine speech examination due to normal speech-language development) made around 36 months of age or 2 years after the time of primary surgery. At the follow-up speech examination, the presence and type of resonance problems and articulatory proficiency were determined based on patients' speech samples. A speech-language pathologist in the clinic measured perceptual judgments of the severity of hypernasality and articulatory proficiency on a seven-point rating scale (1 = normal; 2 = minimal; 3 = mild; 4 = mild to moderate; 5 = moderate; 6 = moderate to severe; 7 = severe).

### 2.3. Statistical Analysis

Dependent variables included (1) rate of occurrence oronasal fistula, (2) percentage of patients who had received secondary palatal surgery for VPI, (3) percentage of patients receiving speech therapy as recommended, (4) percentage of patients demonstrating significant hypernasality above mild to moderate, and (5) percentage of those exhibiting articulatory problems above mild to moderate requiring speech therapy. Chi-square analyses were performed to examine the effect of cleft type on the dependent variables.

## 3. Results

### 3.1. Total Patients

This study included 292 patients with nonsyndromic overt cleft palate (±cleft lip) who received primary palatal surgery at the Seoul Asan Medical Center between 2007 and 2012. The patients included 147 boys and 145 girls and ranged in age from 36 months to 19 years 4 months (mean = 72.48 months) at the time of data collection. Fourteen percent of the total patients had UCLP, 32.2% had BCLP, and 53.8% had CPO. The patients ranged in age at primary palatal surgery from 9 months to 5 years and 3 months, with the mean age of 11.97 months.

Table 1 shows the rate of oronasal fistula following primary palatal surgery, the percentage of patients who had received secondary palatal surgery for VPI, the percentage of patients who received speech therapy as recommended, and speech outcomes at 36-month follow-up for all patients by type of cleft. The retrospective analysis showed that 7.9% (*n* = 23) of all patients developed oronasal fistula following primary palatal surgery. Furthermore, 19.2% (*n* = 56) of the total group had received secondary palatal surgery for VPI, and the mean age for the surgery was 63.7 months (range = 35 months to 14 years and 9 months). Finally, 50.3% (*n* = 147) of all patients were enrolled in or had previously received speech therapy. Regarding speech results at 36-month follow-up, 28.8% (*n* = 84) of the total group demonstrated significant hypernasality, obtaining a score of 4 points or greater on 7-point scale. In addition, 51.4% (*n* = 150) showed significant articulatory deficits of a degree of severity greater than mild, requiring direct speech therapy.

### 3.2. The Effect of Cleft Type on Clinical Outcomes

Patients with CPO appear to have a slightly higher rate of oronasal fistula development following primary palatal surgery (9.6%) compared to patients with UCLP or BCLP (7.3% and 5.3%, resp.). However, Chi-square analyses revealed that the rate of oronasal fistula was not significantly different among the three groups of cleft type. The relationship between cleft type and percentage of patients who had received secondary palatal surgery was significant (*χ* ^2^ = 11.0, *P* = .004). A relatively higher percentage of patients with UCLP or BCLP (26.8% and 27.7%, resp.) received secondary palatal surgery for VPI compared to patients with CPO (12.1%). A significant relationship was also evident between cleft type and percentage of patients who were currently enrolled in or had previously received speech therapy (*χ* ^2^ = 18.4, *P* < .001). The percentage of the patients with UCLP or BCLP (68.3% and 61.7%, resp.) who received speech therapy was higher compared to that of patients with CPO (38.9%). The results showed a significant relationship between cleft type and percentage of patients demonstrating a degree of hypernasality greater than mild (*χ* ^2^ = 29.3, *P* < .001). Consistent with the results of secondary palatal surgery for VPI, a relatively higher percentage of patients with UCLP or BCLP (36.6% and 44.7%, resp.) demonstrated significant hypernasality compared to patients with CPO (17.2%). In addition, a significant relationship was evident between cleft type and percentage of patients showing significant articulatory deficits of a degree of severity greater than mild (*χ* ^2^ = 17.5, *P* = .002). The percentage of patients with UCLP and BCLP who demonstrated significant articulatory deficits (70.7% and 55.3%, resp.) was higher compared to that of patients with CPO (43.9%), which was consistent with the results of the relationship between cleft type and percentage of patients who were currently or previously enrolled in speech therapy.

## 4. Discussions

This study performed retrospective analysis of clinical outcomes of 292 patients with nonsyndromic overt cleft palate (±cleft lip) who received primary palatal surgery at the Seoul Asan Medical Center between 2007 and 2012. This study focused on three postoperative outcomes, the rate of oronasal fistula and the rate of VPI based on the percentage of secondary palatal surgery as well as speech outcomes related to hypernasality and articulatory proficiency. This review indicated that 7.9% of all patients developed oronasal fistula following primary palatal surgery. The literature has reported various rates of oronasal fistula, ranging from 0% to 12.8% in the recent studies [5, 7–12]. These results could be attributed to different inclusion criteria for oronasal fistula found in the literature. In addition, the incidence of oronasal fistula may be closely related to several factors, including cleft type, width of cleft, surgical techniques, and preoperative orthopedics. Among the influential factors, this study examined the effect of cleft type on the incidence of oronasal fistula. We found no significant effect of cleft type on the rate of oronasal fistula. In general, the literature reports that patients with complete bilateral clefts (Veau IV) are more likely to develop a fistula [10, 11]. In this study, patients with UCLP or BCLP had a relatively lower rate of oronasal fistula development following primary palatal surgery compared to patients with CPO, although the results did not reach statistical significance. The low incidence of oronasal fistula in patients with UCLP or BCLP in this study might be related to preoperative NAM. Approximately 90% of babies born with UCLP or BCLP in our institution were treated with preoperative NAM, but only few babies with CPO underwent preoperative procedure. Recently, preoperative orthopedics, such as the NAM procedure, was reported to contribute to the narrowing of the cleft width and therefore reducing the rate of oronasal fistula developed following primary palatal surgery [10, 17, 18]. However, we could not obtain valid medical records on NAM, as the preoperative procedure was performed at a local dental clinic outside the institution. Future research should use objective and systematic data to examine whether patients who received the preoperative orthopedic procedure show better clinical outcomes after primary cleft repair.

The study also reported that 19.2% of the total group had received secondary palatal surgery for VPI. The incidence rates of persistent VPI following primary surgery also vary in the literature, being as high as 30% [5–7, 13–15]. Such varied outcomes concerning VPI following primary surgery might be associated with several factors, including variability in the definition of or criteria defining VPI, different surgical techniques, cleft type, and extent of clefting. Although many studies used mainly secondary corrective surgery as inclusion criteria of VPI, some studies included results evidenced by perceptual assessment of hypernasal speech and assessments using nasoendoscopy and/or videofluoroscopy. Regarding hypernasality, which is a speech problem associated with VPI, 28.8% of the total group demonstrated significant hypernasality requiring secondary palatal surgery or speech therapy. In general, secondary palatal surgery has been performed at our institution in cases where anatomic deficits of the velopharyngeal mechanism following primary repair appear to show persistently moderate or severe degrees of hypernasality. Patients who exhibit a mild to moderate degree of hypernasality and simultaneous articulatory deficits are referred to speech therapy. Therefore, this clinical decision-making process results in differences between the percentage of patients demonstrating significant hypernasality and the rate of secondary palatal surgery. In addition, the results showed that cleft type has a significant effect on the incidence of VPI, that is, the percentage of patients who had received secondary palatal surgery. A relatively higher percentage of patients with UCLP or BCLP received secondary palatal surgery for VPI compared to patients with CPO.

The percentage of patients enrolled in speech therapy or demonstrating significant articulatory deficits requiring speech therapy was high in this study. Approximately half of all the patients had enrolled in or received speech therapy, and 51.4% showed significant articulatory deficits. These results of speech outcomes in this study were consistent with the previous study [6], which examined speech production of preschoolers with cleft palate. Hardin-Jones and Jones concluded that the majority of preschoolers with cleft palate continue to demonstrate speech problems that require direct speech therapy, despite advances in management of cleft palate [6]. A significant relationship was also found between cleft type and speech outcomes. More patients with UCLP and BCLP were enrolled in speech therapy and more demonstrated significant articulatory deficits compared to patients with CPO. This result might be associated with the relatively higher rate of VPI in patients with UCLP and BCLP compared to those with CPO. That is, more patients with UCLP and BCLP showed significant hypernasal speech and articulatory problems due to VPI and therefore needed to receive speech therapy.

To implement a better clinical service approach, it is important to evaluate complications and surgical outcomes. It is also necessary to identify factors that influence surgical outcomes and successful clinical management of individuals with cleft palate. Clinical outcomes of primary cleft palate repair are related to several factors, including cleft type, the extent of innate clefting, surgical repair techniques, expertise of the operating surgeon, preoperative orthopedics, and timing of primary palatal repair. This study examined the effect of only one influential factor on clinical outcomes. Future research should investigate the relationship between several influential factors and clinical outcomes comprehensively.

A final comment on the limitations of this study appears warranted. Several limitations arose in the retrospective review of patient records, which is subject to confounding factors. Especially, the limitation was evident in classifying cleft type and comparing the results with those of previous studies. We found that the cleft classification system of patients' electronic medical records was not consistent and sometimes insufficient, as several junior doctors were involved in recording patients' cleft type. Information on cleft type generally appeared to be described using the terms such as unilateral (right or left sides), bilateral, complete, and incomplete. However, to classify all patients in the study using the existing medical records, we had to use simple cleft classification system, which does not accurately reflect the magnitude of the defect and does not guarantee the homogeneity of each cleft type group. The classification system in this study, by the nature of its simplicity, may reduce the sensitivity for the analysis of the effect of cleft type on clinical outcomes. More detailed cleft classification system should be used or the exact size or width of clefting should be estimated in future research so that a large degree of heterogeneity in cleft type group can be reduced and the effect of cleft type on clinical outcomes can be sensitively detected. Furthermore, continuous research efforts using prospective analysis should attempt to identify presurgical risk factors for clinical outcomes in primary palatal surgery.

## 5. Conclusion

This study represents clinical outcomes of primary cleft palate repairs based on large data gathered from a single institution and reflects a single surgeon's intensive experience over a 5-year period. This makes it possible to rule out influential and confounding factors, such as expertise of surgeons. The study showed that the incidence of oronasal fistula and the incidence of VPI following cleft palate surgery are comparable to those of other cleft centers worldwide, although care needs to be taken when interpreting the results due to lack of standard definitions used for oronasal fistula and VPI in the literature and the weakness of retrospective analyses. The study also suggests that approximately half of patients show speech problems following primary palatal surgery and require direct speech therapy. This result highlights the importance of routine follow-up speech examinations and multidisciplinary team approach for this population. In addition, the study suggests that cleft type is one of the important factors related to clinical outcomes. This audit provides a retrospective quality review of primary palatal surgery at the Seoul Asan Medical Center and a basis for ongoing research efforts to establish an optimal treatment regimen for patients with cleft palate.

## Figures and Tables

**Table 1:** Clinical outcomes by type of cleft.

Results	UCLP		BCLP		CPO		Total		*χ* ^2^	*P*
*n*	%	*n*	%	*n*	%	*n*	%

Total	41	14.0	94	32.2	157	53.8	292
Oronasal fistula	3	7.3	5	5.3	15	9.6	23
2nd palatal surgery	11	26.8	26	27.7	19	12.1	56
Speech therapy	28	68.3	58	61.7	61	38.9	147
Hypernasality	15	36.6	42	44.7	27	17.2	84
Articulatory deficits	29	70.7	52	55.3	69	43.9	150

UCLP: unilateral cleft lip and palate; BCLP: bilateral cleft lip and palate; CPO: cleft palate only.
